# Botulinum toxin treatment of salivary fistulas following parotidectomy: follow-up results

**DOI:** 10.1007/s10006-012-0375-0

**Published:** 2012-11-24

**Authors:** Rainer Laskawi, Jan Winterhoff, Sabrina Köhler, Laura Kottwitz, Christoph Matthias

**Affiliations:** Department of Otorhinolaryngology, University of Göttingen Medical Center, Robert-Koch-Str. 40, 37075 Göttingen, Lower Saxony Germany

**Keywords:** Parotidectomy, Salivary fistulas, Botulinum toxin, Long-term results

## Abstract

**Background:**

Salivary fistulas are a well-known sequel of parotidectomy, and successful treatment with botulinum toxin has been demonstrated in individual cases. Here, we report on 12 patients with fistulas treated following parotidectomy for various indications.

**Methods and results:**

Injection of botulinum toxin type A into the residual gland tissue was the initial treatment. After *early* intervention (within 6 weeks after development of the fistula), only one fistula remained (9 of 10 fistulas treated early *only* with botulinum toxin). One patient with early intervention did not want to wait for the botulinum toxin treatment to take effect and demanded early surgical revision, which was successful. In one patient with a *permanent* fistula, botulinum toxin treatment began *420 days* after the operation and was unsuccessful. No side effects were evident after the treatment.

**Conclusion:**

In summary, botulinum toxin injections into the parotid tissue remaining after surgery appear to be an effective treatment for salivary fistulas following parotidectomy.

## Background

Salivary fistulas can occur after parotid gland surgery [1, 5, 7, 8, 11, 14, 15]. This occurs in 4 % of the cases in our clinic [8], but incidence rates of up to 14 % have been reported [11, 12]. A number of different therapeutic approaches have been described for the treatment of parotid fistulas subsequent to parotid gland surgery [2, 11, 15]. In recent years, treatment with botulinum toxin (BoNT) has gained increasing recognition [3, 4, 6, 9, 10, 12, 13, 16]. Reports in the literature refer mainly to early fistulas after parotid gland surgery, which had been treated without delay, i.e., before complete wound healing. However, these reports of successful treatment with BoNT are based only on individual case reports [3, 4, 6, 9, 10, 12, 13, 16].

**Fig. 1:**
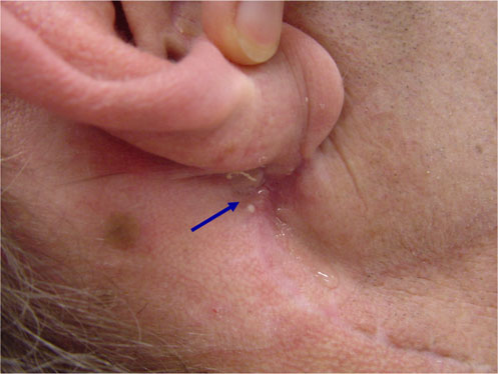
A patient with a right-sided permanent fistula (*blue arrow*). Parotid surgery had been done more than 1 year earlier. Regularly occurring discharge of saliva after gustatory stimuli

Permanent fistulas (see example in Fig. 1) that persist after complete wound healing after the parotid gland procedure are a subgroup that requires special attention. They are difficult to treat [1, 6, 16], and preventive measures should be initiated early on.

This retrospective analysis describes our experience in the treatment of postoperative parotid fistulas with botulinum toxin A. It presents the flow diagram of our current therapy as well as special aspects that have received no mention in the literature, such as the success rate in a larger patient collective.

## Patients and methods

In the period from 2006 to 2011, we treated 12 patients (see Table 1 for characteristics of the cohort) with the clinical diagnosis of a salivary fistula subsequent to surgery on the parotid gland. The fistulas present with repeated leakage of saliva from the caudal, pre-, or subauricular suture in the neck region, particularly after meals. There were seven male and five female patients. The indication for surgery was cystadenolymphoma in seven patients, pleomorphic adenoma in two patients, one parotid cyst, one lymphoma in a HIV-positive patient, and one undefined infiltrative process (explorative parotidectomy). The surgical procedure was a superficial parotidectomy in 11 patients and a partial superficial parotidectomy in one.

**Table 1:** Characteristics of patients included in the study

Number	Age (years)	Disease and kind of surgery	Further complications after parotidectomy	Time interval until development of fistula (days)	Time interval between parotidectomy and first BoNT injection (days)	Dose of each Botox® injection (U)	Closure of fistula after BoNT treatment (yes/no)	Time interval to closure of fistula (weeks)	Further therapy
1	49	Cystadenolymphoma;	Hematoma	2	31	30 + 15	No	-	2× revision surgery, radiotherapy
		s.p.							
2	72	Undefined infiltrative process;	None	10	14	25	Yes	4	–
		s.p.							
3	54	Pleomorphic adenoma;	None	21	23	10	Yes	3	–
		s.p.							
4	58	Cystadenolymphoma;	None	5	10	30	Yes	2	–
		s.p.							
5	56	Cystadenolymphoma;	Sialocele	5	9	30 + 30	?	–	*Early* revision surgery
		s.p.							
6	62	Cystadenolymphoma;	Sialocele	38	43	20	Yes	3	–
		s.p.							
7	32	Cystadenolymphoma;	None	21	29	20	Yes	3	–
		s.p.							
8	44	Pleomorphic adenoma;	None	10	33	40	Yes	2	–
		s.p.							
9	51	Cyst;	Hematoma	17	17	20	Yes	5	–
		s.p.							
10	50	Cystadenolymphoma;	None	15	15	30	Yes	3	–
		s.p.							
11	39	Lymphoma, HIV-positive;	None	14	420	20 + 20	No	-	Radiotherapy
		p.s.p.							
12	61	Cystadenolymphoma;	None	10	10	25 + 15	Yes	5	–
		s.p.							

*s.p.*, superficial parotidectomy; *p.s.p.*, partial superficial parotidectomy

There is no clear definition in the literature of when a salivary fistula subsequent to parotid surgery should be classified as “permanent”. However, clinical experience shows that some patients have a salivary fistula that does not close by itself, which is a challenging starting condition for treatment. Because of the special problems associated with persisting fistulas, we considered it advisable to differentiate between “early fistulas” (BoNT administration earlier than 6 weeks after parotid gland surgery with potentially adaptable wound tissue) and “permanent fistulas” (BoNT administration later than 6 weeks after surgery). According to this classification, our treatment population consisted of 11 patients with “early fistulas” and one with a “permanent fistula”.

As described previously [3], BoNT was injected under ultrasound guidance into the postoperatively remaining glandular tissue to selectively reduce secretory activity. This is similar to the procedure described by other authors [4, 6].

The total dose of BoNT (Botox®, Allergan, 100 units (U) dissolved in 4 ml normal saline) was between 10 and 40 U depending on the size of the remaining glandular compartment. The individual doses were 10 U in one patient, 15 U in two, 20 U in four patients, 25 U in two, 30 U in five, and 40 U in one patient (average dose was 23.3 U). It was injected into the gland at two to three sites. Because of persisting leakage of saliva after the first injection, three patients obtained two injections: 25 and 15 U, 20 and 20 U, and 30 and 15 U, respectively. The time interval between the two injections amounted to 7, 77, and 111 days.

Botulinum toxin A was injected into the remaining tissue between 9 and 420 days after parotidectomy. One patient (number 5 in Table 1) who was injected with 30 U of BoNT 7 days after the appearance of the fistula did not want to wait for the BoNT to take effect and demanded immediate surgical revision of the fistula 42 days after the initial operation. The procedure was performed as wished, although we did not consider it absolutely indicated since wound healing was not yet complete. The fistula was resected under the microscope, and an additional 30 U of Botox® was applied intraoperatively to the remaining glandular tissue.

## Results

The fistulas described in this report appeared between 2 and 38 days (average 14.0 days) after the parotid gland surgery. In 9 of the 12 patients, treatment with BoNT led to a complete closure of the fistula without any further surgical measures. The documented time of closure was 2 to 5 weeks (average was 3.3 weeks) after the BoNT injection.

The flow of saliva to the cheek and neck decreased rapidly and markedly after the injection. No complications due to the BoNT injections were seen.

In one patient (number 1 in Table 1), neither the early injection of BoNT (30 and 15 U) nor the subsequent surgical fistula excision and intraoperative BoNT application were able to close the fistula. Radiation therapy (total dose of 30 Gy) and renewed excision were ultimately successful [1]. The fistula of the patient (number 5 in Table 1) who did not want to wait for the potentially positive effect of the BoNT injection was closed by microsurgical fistula extirpation and intraoperative BoNT injection.

The treatment of a patient (number 11 in Table 1) with a persisting fistula with BoNT injections 420 days after parotidectomy (20 units and 20 units) and surgical revision was unsuccessful. Because of the great emotional strain, he was given low-dose radiation therapy that was eventually successful.

In summary, the data presented here with their special aspects show that salivary fistulas were healed in 9 of 10 patients (90 %) with BoNT treatment alone when treatment started early after the development of the fistula and time was allowed for the treatment to take effect. Treatment was unsuccessful in only one patient of this group.

## Discussion

Salivary fistulas are a well-known complication that can occur after parotid gland surgery. Numerous medical and surgical interventions have been described as therapeutic options in the literature [11, 12]. The most important therapeutic principle is to reduce the secretion of the remaining glandular tissue in order to both alleviate the symptoms and facilitate closure of the fistula. This goal can be attained in a minimally invasive manner with botulinum toxin.

There was a 90 % rate of successful fistula closures (9 of 10) in this retrospective analysis of early-onset BoNT therapy. Our current therapeutic approach as described here in a larger number of patients confirms previous observations of other authors [3, 4, 6, 9–12, 16]. The results could indicate that the local conditions for fistula healing are improved by the reduction of salivary production, and closure of the fistula may only have become possible due to the application of BoNT.

In addition, reducing the volume of the aggressive agent “saliva” produced in the remaining glandular tissue might allow the fistula to heal earlier, i.e., the treatment might affect the time factor. In the present analysis, closure of the fistulas was achieved in the time frame of 2 to 5 weeks. In this study, we described a cohort of 12 patients, and it is important to state that a larger study with more patients with this rare complication will be helpful in the future. In this connection, it will be of interest to perform such a study including a matched untreated group with spontaneous closure.

The reduction of salivary flow that occurs within a few days after the injection as described in numerous publications [4, 6, 9, 10, 12, 16] must not be equated with the end of wound healing. Nevertheless, this is a very important effect since it alleviates the patients’ symptoms and reduces the discomfort associated with saliva draining through the fistula and running down the cheek and neck. Even if fistula closure cannot be achieved, this is still a very positive effect of the BoNT injection since it eliminates the visible social stigma.

Improvement is also possible with permanent fistulas even if fistula closure cannot be achieved. Guntinas and Sittel reported that salivary flow from a permanent fistula ceased for up to 11 months after BoNT application [4]. This indicates that BoNT is at least a rapidly acting symptomatic treatment.

There are, at present, no standardized dose recommendations for this indication, but the doses we used showed an adequate effect. They were relatively high compared to the dose normally used to treat hypersalivation (22.5 U of Botox® for each gland, ref. [3]) considering the reduced size of the glandular compartment. Lim and Choi [9] point out that lower doses of BoNT are effective if the substance is correctly injected into the glandular tissue. This was ensured in our patients by the ultrasound-guided injection technique [3]. It should also be noted that our chosen dosage is similar to that described by other authors [6, 9, 10, 12].

In summary, our experience shows that when dealing with fistulas subsequent to parotidectomy, it is therapeutically relevant to distinguish between early and permanent fistulas. Treatment of parotidectomy-associated salivary fistulas with botulinum toxin A should commence early. It is our opinion that the most important aspect of BoNT therapy is the reduction of saliva production in the remaining glandular tissue as this seems to have a positive effect on the healing process. The resulting potential shortening of the healing period and the confirmed immediate reduction of saliva drainage over the parotid region and neck [3, 4, 6, 9, 10, 12, 13, 16] are great advantages for the patient with regard to local side effects and the social stigma. This argues for the early use of BoNT, regardless of an ultimate closure of the fistula. Our observations show that there is a good chance of healing (9 out of 10, 90 %) after early treatment with BoNT.

Permanent fistulas pose special problems for treatment [1, 6, 16]. Radiation therapy should be considered as a last resort [2].

**Fig. 2:**
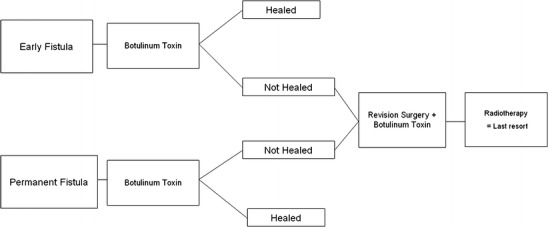
Our therapy regimen for the treatment of salivary fistulas. The strategy shown here is aimed at fistula closure

The flow diagram in Fig. 2 illustrates our current therapeutic approach. We agree with Marchese-Ragona et al. [11, 12] that further prospective studies with BoNT with a greater number of patients should be conducted to confirm and optimize the therapy with regard to effective dose and timing.
